# OryzaPG-DB: Rice Proteome Database based on Shotgun Proteogenomics

**DOI:** 10.1186/1471-2229-11-63

**Published:** 2011-04-12

**Authors:** Mohamed Helmy, Masaru Tomita, Yasushi Ishihama

**Affiliations:** 1 Institute for Advanced Biosciences, Keio University, 403-1 Daihoji, Tsuruoka, Yamagata 997-0017, Japan; 2 Systems Biology Program, Graduate School of Media and Governance, Keio University, 5322 Endo, Fujisawa, Kanagawa 252-0882, Japan; 3 Graduate School of Pharmaceutical Sciences, Kyoto University, Sakyo-ku, Kyoto 606-8501, Japan

## Abstract

**Background:**

Proteogenomics aims to utilize experimental proteome information for refinement of genome annotation. Since mass spectrometry-based shotgun proteomics approaches provide large-scale peptide sequencing data with high throughput, a data repository for shotgun proteogenomics would represent a valuable source of gene expression evidence at the translational level for genome re-annotation.

**Description:**

Here, we present OryzaPG-DB, a rice proteome database based on shotgun proteogenomics, which incorporates the genomic features of experimental shotgun proteomics data. This version of the database was created from the results of 27 nanoLC-MS/MS runs on a hybrid ion trap-orbitrap mass spectrometer, which offers high accuracy for analyzing tryptic digests from undifferentiated cultured rice cells. Peptides were identified by searching the product ion spectra against the protein, cDNA, transcript and genome databases from Michigan State University, and were mapped to the rice genome. Approximately 3200 genes were covered by these peptides and 40 of them contained novel genomic features. Users can search, download or navigate the database per chromosome, gene, protein, cDNA or transcript and download the updated annotations in standard GFF3 format, with visualization in PNG format. In addition, the database scheme of OryzaPG was designed to be generic and can be reused to host similar proteogenomic information for other species. OryzaPG is the first proteogenomics-based database of the rice proteome, providing peptide-based expression profiles, together with the corresponding genomic origin, including the annotation of novelty for each peptide.

**Conclusions:**

The OryzaPG database was constructed and is freely available at http://oryzapg.iab.keio.ac.jp/.

## Background

Among high-throughput experimental methods, genome sequencing represents a turning point in the understanding of biological systems. Nevertheless, the biological significance of the sequenced genome cannot be understood unless the protein-coding genes and their products are accurately identified. Thus, genome annotation has become major issue [1-3]. Genome annotation is the process of gene structure and function determination, and it usually takes place after genome sequencing and before data deposition in a database or databank [2,4,5].

In typical genome annotation work, experimental and computational methods are integrated to analyze the huge volume of sequence data [2,4,6,7]. Thus, genome annotation is highly dependent on the expression evidence, usually transcriptional, provided by experiments and the algorithms implemented in the computational tools [8]. Consequently, the annotation process suffers from several limitations. For instance, most of the sequenced genomes lack rich transcriptional evidence, e.g., a full-length cDNA library. Even when such information is available, evidence of expression at the transcriptional level does not necessarily imply translation into a protein [8,9]. Therefore, annotation is highly reliant on *de novo *annotations of protein-coding genes performed using gene prediction programs [2,4,8].

On the other hand, gene/protein prediction tools have proven their usefulness and utility in the annotation process. However, the prediction accuracy varies from one tool/algorithm to another and from one organism to another, depending on the genome complexity [2,8,10,11]. For instance, in the human and *Arabidopsis *genomes, the prediction accuracy amounted to 50% and ~66%, respectively, indicating the need for better identification and validation methods [11,12].

Mass spectrometry-based proteomics, as an experimental approach to measure proteins, can provide translation-level expression evidence for the predicted protein-coding genes; this is the so-called proteogenomics approach of using large-scale proteome data in genome annotation refinement [3,8,13,14]. This approach seems the best option for identification and validation of protein-coding genes, or at least a significant portion of them, in an independent and unambiguous way. This can be achieved by detecting the naturally occurring proteins (proteomics) and systematically mapping them back to the genome sequence (genomics) [3,8,13,14]. In addition to validating predicted gene models at the translation level [15,16], proteogenomics has other useful applications, such as finding new gene models [7], determination of protein start and termination sites [17], finding and verifying splice isoforms at the protein level [18] and verification of hypothetical and putative genes/proteins [17,19]. The results of proteogenomic studies are usually made freely available via specialized databases such as AgBase [20] or are included in databases developed particularly to host data from specific projects, such as the AtProteome database developed to host the *Arabidopsis *proteogenomics data [21]. Overall, proteogenomics represents a promising approach for application to both completed and newly sequenced genomes.

Rice (*Oryza sativa*) is one of the most important food crops; almost half of the world's population is estimated to rely totally or partially on it. Moreover, rice considered a model organism because of its relatively small genome (12 chromosomes and ~370 Mbp) [22,23]. The whole genome sequence and annotation have been published and updated several times (5 builds for the genome and 6 builds for the annotation to date) [24-26]. However, there has been little attempt to include proteome information in the genome-wide annotation, except for the work of Itoh and colleagues, who used rice proteome data, available through the rice proteome database [27], to confirm 834 ORFs [25]. The virtual absence of proteome-based genome annotation for rice is possibly due to the absence of accurate and detailed rice proteome information.

Here we present (OryzaPG-DB) a rice proteome database based on shotgun proteogenomics. Unlike the currently available rice proteome database [27], which provides the 2D-PAGE-based proteome, OryzaPG-DB contains peptides obtained from shotgun-based proteomics with their product ion spectra, as well as updated annotations, side by side with the corresponding protein, cDNA, transcript and genomic sequences and information.

## Construction and content

### Generation of a reference dataset by shotgun proteomics

**Figure 1:**
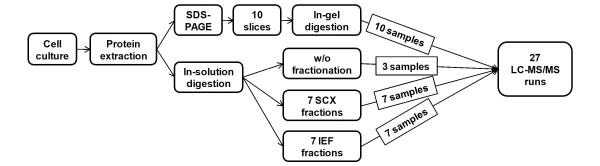
**The experimental workflow to obtain the rice proteome**. Proteins from undifferentiated cultured cells were extracted, digested, and pre-fractionated, and 27 samples were prepared for the subsequent LC-MS/MS analysis.

To perform proteogenomics-based genome annotation refinement of rice, we firstly generated a dataset by using a shotgun proteomics approach with an LTQ-orbitrap high-accuracy mass spectrometer for analysis of undifferentiated cultured rice cells (see supplementary methods in additional file 1). The experimental workflow is shown in Figure 1. Three pre-fractionation procedures, SDS-PAGE at the protein level, and strong cation exchange chromatography (7 runs) [28] and isoelectric focusing (7 runs) [29] at the peptide level, were employed prior to nanoLC-MS/MS analysis to extend the proteome coverage. As a result, 156,871 product ion spectra were acquired from 27 LC-MS/MS runs. Undifferentiated cultured rice cells were selected as the first sample to construct our bioinformatics pipeline and data repository system.

**Figure 2:**
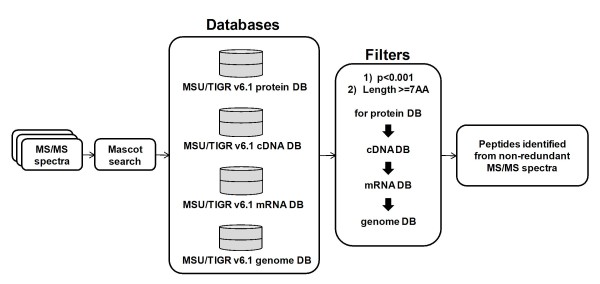
**Informatics analysis flowchart to create list of peptides/proteins identified from non-redundant product ion spectra**.

Regarding the databases for MASCOT [30] search, the Rice Genome Annotation Project, currently at Michigan State University (MSU), offers rice protein, cDNA, transcript and genome databases (MSU DB) as *de novo *annotation of the rice genome sequence, with further improvements and modifications using full-length cDNA and EST alignment [26] Thus, we decided to use these four databases for peptide/protein identification, since each of them provides a special opportunity to identify novel peptides. The protein database provides all potential protein and peptide sequences. The cDNA database is similar to the protein database, but allows searching six-frame translations of the nucleotide sequence, and therefore, it is possible to identify exon-exon junction peptides and exon-skipping events from the two databases [18,31]. The transcript database includes introns, so we can identify exon-intron spanning and intronic peptides. Finally, the genome database includes the intergenic regions, offering the potential to find new non-annotated genes. Thus, each of the four databases affords specific search advantages (Figure 2).

As a result, we identified 14,955 unique peptides from the database searching against MSU protein database (V6.1). Further, we identified 166 additional unique peptides from three nucleotide sequence databases (cDNA, transcript and genome databases). Then, the peptides obtained in this study were mapped to the annotated MSU genome (V6.1) to identify proteins (cDNAs) and unspliced mRNAs (genes) (Table 1). Genes with peptides mapped to novel regions such as intron, exon-intron boundary and non-coding region, are counted as "Genes to be revised", indicating that the annotation of these genes needs revision.

**Table 1:** Summary of the currently available contents in OryzaPG-DB*

Item	Current contents**	Remarks
Proteins/cDNAs	5,034	Corresponds to MSU/TIGR (6.1) models
Genes/us-mRNAs	3,182	Corresponds to MSU/TIGR (6.1) locus/transcript (TU)
Total peptides	15,121	All identified non-redundant peptides
Novel peptides	166	Peptides that are not present in the protein database
Genes to be revised	40	Genes with peptides mapped to novel regions such as intron, exon-intron boundary and non-coding regions

* Values in this table are statistics of the database contents on the launching date. All updates can be found in the UPDATES page in OryzaPG-DB website.

** On 22/Feb/2011.

### Proteogenomics analysis to find novel genomic features

Next, we performed proteogenomic data analysis using bioinformatics approaches to map the identified peptides back to the genome and find novel genomic features as follows:

▪ Download the original annotation from MSU genome browser with only the MSU Osa1 Rice Gene Models and MSU Osa1 Rice Loci features selected. The original files can also be obtained from the OryzaPG-DB download page.

▪ Align all peptides identified from the MSU protein, cDNA and transcript databases to their corresponding genomic origin (genomic-unspliced mRNA), using the Basic Local Alignment Search Tool (BLAST) [32]. The alignments were performed using a local version of NCBI BLAST (blast2seq) [33] and perl script.

▪ Extract the alignment results of the peptides identified from the MSU genome database directly from MASCOT output files.

▪ Create perl scripts that read the alignment results and convert them to standard GFF3 format. Each peptide's alignment was converted to a GFF3 line indicating its type, identification source, start, end, parent and OryzaPG-DB peptide ID.

▪ Map the peptides identified from the MSU genome to the genes by comparing the peptide alignment coordinates (start and end) with the gene coordinates. If a peptide's start and end are between or overlapping with a gene's start and end, we map this peptide to that gene and create a GFF3 line similar to the one described above.

▪ Update the original annotation files by appending the peptides' GFF3 lines obtained from our analysis to the end of the corresponding gene. So far, we have created an updated annotation in GFF3 format containing the original annotation and the proteome information. Thus, the "Type" column in the updated GFF3 files includes the type "peptide" beside the original types (3'UTR, 5'UTR, CDS,...etc). However, the identified novel peptides require further analysis to find out whether or not they represent novel genomic features.

### Peptide novelty assessment and visualization of gene features

**Figure 3:**
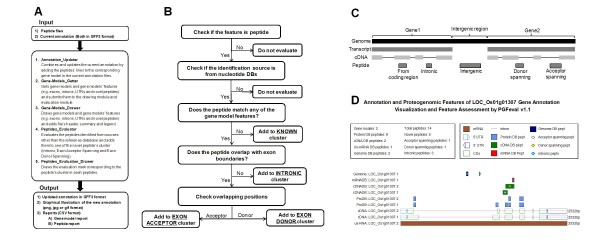
**Assessment and visualization of a peptide's genomic novelty**. (A) The design and architecture of PGFeval. (B) The assessment algorithm used in evaluating the peptide's novelty in PGFeval. (C) Schematic illustration of peptide clusters. (D) Example of the graphical output of PGFeval.

The genomic features can be visualized using tools such as the generic genome browser (Gbrowse) or UCSC genome browser [34,35], but determination of whether or not the peptide represents a novel genomic feature and the type of novelty, e.g., intronic or exon-boundary spanning, cannot be done with these tools. We consider a peptide novel if it does not exist in the protein database. Therefore, all the peptides identified from the other three databases are considered novel. However, this does not mean that such a peptide represents a novel genomic feature. The peptide may be aligned to a known coding region, but may not exist in the protein database, due to incompleteness [36,37]. Hence, we need an evaluation tool and algorithm to assess the genomic novelty of each novel peptide. Therefore, we developed PGFeval (ProteoGenomic Features Evaluator), an evaluation and visualization tool using perl and the GD library http://www.libgd.org, which evaluates the genomic novelty of each peptide and draws the whole gene model with graphical annotation that incorporates the genomic novelty of the peptides. PGFeval analyzes the updated annotation file in GFF3 format and uses the type, start and end to draw the gene and its structural elements, such as the UTRs and exons (Figure 3A). Next, it implements an assessment algorithm to evaluate and cluster the peptides into four clusters (intronic, exon acceptor spanning, exon donor spanning and known) (Figure 3B). The four clusters are illustrated in Figure 3C. In addition, PGFeval exports two CSV reports, a genes report and a peptides report, in a master-slave style. The genes report contains one entry per gene summarizing the gene's features such as total peptides, number of novel peptides and novel genomic features, while the peptide report contains one entry per peptide, indicating its gene and assessment result, such as novelty, cluster and identification source. Thus, the two reports can be easily analyzed using any spreadsheet software or imported into any relational database as two tables with one-to-many relationship. Figure 3D shows an example of the PGFeval graphical output.

This analysis revealed 51 new genomic features in 40 genes. The majority of the novel features consisted of intronic peptides (36), while the exon boundary-spanning peptides consisted of 13 donor-spanning and 2 acceptor-spanning peptides. The remaining novel peptides were mapped to known coding regions.

### Generic scheme design for the relational database

**Figure 4:**
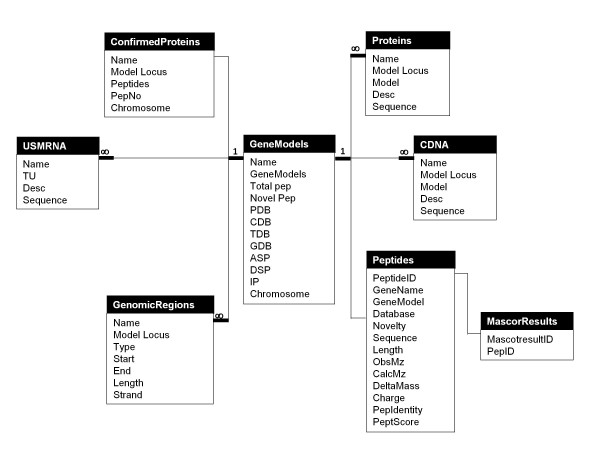
**OryzaPG database scheme**.

We attempt to design a generic and simple database scheme that would be suitable for such proteogenomic data and that could be used in similar shotgun proteogenomics projects. As long as the annotation main unit is the gene, in our design (Figure 4), the database main entity is the gene as well. We used the MSU V6.1 locus as our gene ID, joining all other gene components together. All other database entities, such as protein, cDNA, unspliced mRNA and peptide, take the gene ID as a foreign key. Further, protein, cDNA, and un-spliced mRNA have their own IDs and aliases that were used in MSU V6.1. Peptide IDs start with P, C, T or G, which indicates that the identification source of the peptide is the protein, cDNA, transcript or genome database, respectively. The letter is followed by the serial number of the peptide in its sample, *e.g.*, P345 is the peptide number 345 among the peptides identified from the protein database. The peptide ID also joins the peptide with its MASCOT results page and product ion spectral details. In the updated annotation files, the peptides are assigned to their parents using the notation "parent ID:peptide ID" e.g. LOC_Os06g01230.1:P62531.

### Database implementation and web interface development

As mentioned above, PGFeval exports two reports: genes report and peptides report. Both reports are designed in master-slave style. Thus, both were imported directly into the database. The protein, cDNA, and transcript information such as the IDs, aliases, descriptions, lengths and sequences were extracted from the FASTA files and the GFF3 files obtained from the MSU website and MSU genome browser, then converted to tables using perl scripts. Next, HTML files, similar to MASCOT peptide view files, were generated and imported into the OryzaPG-DB server. The data were later imported into a database implemented using the MySQL server. The annotation files (GFF3) and the visualization files (PNG) are stored in the web server directly. The whole system is thus a two-tier web-based system.

The web interface was developed using HTML, Java script and the server side scripting was done using PHP. The database was implemented using the MySQL server. We host the system on Microsoft Internet Information Service (IIS V7.5) on a Dell server running Windows 7 at the Institute for Advanced Biosciences (IAB), Keio University.

### OryzaPG-DB Application Programming Interfaces (APIs)

The application programming interface (API) is an interface implemented by the application to allow interaction with the operating system or other programs. An API determines the protocol and parameters required to run certain functions or parts of the program and to return the results of its execution [38].

In OryzaPG-DB, we provide users with several URL APIs for data retrieval. For each entity, we provide users with an API that returns the results per record, per chromosome or for the whole genome. The data are returned in tabular view or in FASTA format with minimum formatting to allow easy processing. The complete list of the available APIs and their parameters can be found on OryzaPG-DB API Guide, available in the OryzaPG-DB website.

## Utility and Discussion

### Shotgun proteogenomics

The current rice genome annotation includes 56,797 genes, of which most are either putative (23,348 genes) or hypothetical (8,885 genes) or conserved hypothetical (2,003 genes) [26], http://rice.plantbiology.msu.edu]. Thus, the total number of genes for which experimental expression evidence is lacking represents more than 60% of the total annotated genes. Moreover, the available expression evidence (for 6,311 genes, representing about 10% of the total) is based on transcription, which does not necessarily imply translation to protein [8]. This indicates the need for a novel approach to improve and refine the rice genome annotation.

To perform genome annotation refinement of rice by means of a proteogenomics approach, we firstly need accurate and high-throughput proteome information. Thus, we generated the rice MS/MS-based proteome using our highly accurate nanoLC-MS/MS proteomics facility (see additional file 1). We started with undifferentiated cultured rice cells to generate data for the construction of our bioinformatics pipeline and data repository system, because a relatively unbiased expression profile of the rice proteome was expected, based on the report that an *Arabidopsis thaliana *proteomics study using cultured cells covered over 70% of the differentiated organ proteome [21]. We plan to generate similar datasets for all vegetative organs throughout the rice life cycle (see future work).

The generated data were compared against four databases (protein, cDNA, transcript and genome) for peptide/protein identification and the resultant peptides were filtered using Mascot score and peptide length to select peptides with high identification confidence and high specificity (p value < 0.001 and false-positive rate (FPR) < = 1%). Then, the peptides identified from the protein database together with the novel peptides identified uniquely from the other three databases were used to create list of peptides identified from non-redundant product ion spectra.

We utilized these peptides to perform proteogenomic analysis for the rice genes within our sample coverage. Our analysis revealed novel genomic features in 40 genes. In addition, 112 peptides, from the genome database-identified peptides, were mapped to intergenic regions, indicating the possible existence of non-annotated genes.

### OryzaPG-DB Utility

**Figure 5:**
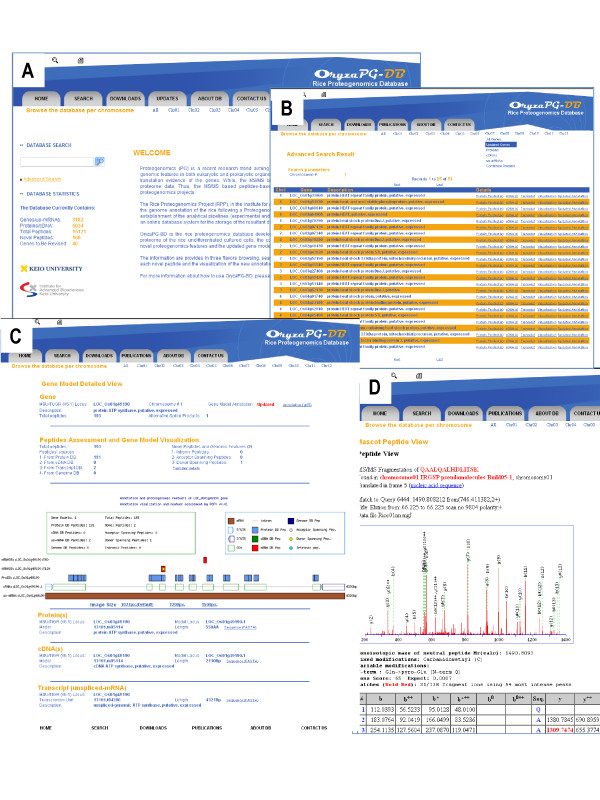
**OryzaPG screenshots**. (A) The advanced search form. (B) Example of advanced search results. (C) Gene detailed view including information on the gene, its protein products, cDNAs, transcripts and graphical visualization. D) Peptide view and the product ion spectra. The browse bar at the top of all pages allows the user to navigate through the database displaying data for all chromosomes or per chromosome.

OryzaPG-DB provides the scientific community with the first high-throughput MS/MS-based rice proteome information and proteogenomic results, publicly available through a comprehensive web interface (Figure 5). The web interface provides several means of data acquisition. Users can browse the database displaying data for genes, updated gene models, proteins, cDNAs and transcripts for the whole genome or per chromosome. In each case, the details of each record will be displayed together with links to related products, genomic origin and identified peptides. In addition, the sequence in FASTA format, the annotation in GFF3 format and visualization in PNG are available for download per displayed record. Further, external links to other databases such as NCBI, MSU and RAP are available. Users can also search the database using keyword search or parameter search, and the search results are displayed per gene. The download page provides users with all the data generated in this project and the data used to perform the proteogenomic analysis. Links to the websites that contains the original datasets and instructions for how to download the original annotations are also provided.

### Future work

Plans for further development of OryzaPG-DB are mainly focused on the content and consequently also the interface. We plan to extend the data to include rice root, stem, leaf blade and other organs as soon as we generate those proteomes. In addition, proteogenomic analysis will be available for all genes covered by the new samples. The interface, therefore, will be updated to allow browsing the data by sample, organ, etc., and we will also add advanced search parameters, enabling auto-generation of updated FASTA sequences using experimentally based genome re-annotation.

## Conclusions

The rapid growth of available sequenced genomes requires novel approaches to identify genes and their functions, as well as sustainable data repository systems to store the accumulated data and make it publicly available for researchers. Proteogenomics is a novel approach combining MS/MS-based proteomics with genomic information and bioinformatics to enhance genome annotation. In this report, we present OryzaPG-DB, the data repository system of the Rice Proteogenomics Project. OryzaPG-DB provides interested rice biologists with the MS/MS-based proteome and the results of proteogenomic analysis, together with all the genomic information within our coverage. The database currently contains the results for cells from undifferentiated culture, and it is planned to be updated periodically with the results of analysis of samples from all vegetative organs of rice. We believe OryzaPG-DB will be an important resource and data-serving tool for rice biologists.

## Availability and Requirements

OryzaPG-DB is freely available at http://oryzapg.iab.keio.ac.jp. In the development of Oryza-PG DB, we followed the usual standards of web applications development and the Java scripts employed are cross-browser scripts. We have confirmed that OryzaPG-DB is fully functional on four web-browsers, Google Chrome, Mozilla Firefox, Microsoft Internet Explorer and Safari, in five operating systems, Windows XP, Vista and 7, Linux Ubuntu and Mac OS (10.5), with no need for any plug-ins or special system requirements.

## Authors' contributions

YI designed the experiments and coordinated the whole project. MH performed the experiments, bioinformatics analysis, designed and developed PGFeval, the database and the web interface. MT supervised the project. MH wrote the first draft of the manuscript. All authors read and approved the final manuscript.

## Supplementary Material

Additional file 1 **Supplementary Materials and Methods**. Details of materials and experimental procedures used to produce the utilized dataset. Click here for file
