# Supplementary material for: OryzaPG-DB: Rice Proteome Database based on Shotgun Proteogenomics
Source: BMC Plant Biol. 2011 Apr 12;11:63. doi: 10.1186/1471-2229-11-63 (PMC3094275; doi:10.1186/1471-2229-11-63)
Supplement: Additional file 1 — Supplementary Materials and Methods. Details of materials and experimental procedures used to produce the utilized dataset. [file 1471-2229-11-63-S1.DOC]

OryzaPG-DB: Rice Proteome Database based on Shotgun Proteogenomics

by Mohamed Helmy, Masaru Tomita, Yasushi Ishihama

**Additional File 1**

**Supplementary Materials and Methods**

***Sample Preparation***

*In-gel digestion*

Rice cultured cells (~180 mg) were frozen in liquid nitrogen and then disrupted with a Multi-beads shocker (MB400U; Yasui Kikai), as previously reported . SDS-PAGE was performed using Wako Super Sep Ace precast gel (Osaka, Japan). The gel was stained with negative gel stain MS kit (Wako), and sliced into 10 slices, followed by in-gel digestion as described . These digested samples were desalted using StageTips with C18 Empore disk membranes (3 M) for the subsequent LC-MS/MS analysis.

*In-solution digestion*

The same disrupted cells were suspended in 0.1 M Tris–HCl (pH 9.0), transferred to the homogenizer and homogenized. Then, the homogenate was reduced with dithiothreitol, alkylated with iodoacetamide, and digested with Lys-C, followed by dilution and trypsin digestion as described . These digested samples were desalted using StageTips with C18 Empore disk membranes (3 M). The peptide concentration of the eluates was adjusted to 1.0 mg/ml with 0.1% TFA and 80% acetonitrile. The resultant peptides were pre-fractionated using two methods, strong cation exchange (SCX) (7 fractions) and isoelectric focusing (IEF) (7 fractions) , resulting in 14 peptide samples, which were evaluated by LC-MS/MS.

***Peptide pre-fractionation***

*SCX pre-fractionation*

Pre-fractionation of digested peptides was carried out according to the StageTips fractionation protocol , with a strong cation exchange (SCX) disk (SCX-StageTips ). Elution with 20-500 mM ammonium acetate solutions containing 15% acetonitrile afforded five fractions. Eluates, including the flow-through fraction, were desalted with C18-StageTips.

*IEF pre-fractionation*

Another portion of the in-solution-digested peptides was pre-fractionated by use of the IEF pre-fractionation method with a ZOOM® IEF Fractionator (Invitrogen) according to the manufacturer’s protocol. Digested peptides (890 µg) were loaded onto the ZOOM® IEF Fractionator, and five IEF fractions, enriched in peptides with p*I* values of 3–4.6, 4.6–5.4, 5.4–6.2, 6.2–7, and 7–10, were obtained.

***LC-MS/MS analysis***

An LTQ-Orbitrap XL (Thermo Fisher Scientific, Bremen, Germany) coupled with a Dionex Ultimate3000 pump (Germering, Germany) and an HTC-PAL autosampler (CTC Analytics AG, Zwingen, Switzerland) was used for nanoLC-MS/MS analyses. A self-pulled needle (150 mm length × 100 µm I.D., 6 µm opening) packed with ReproSil C18 materials (3 µm, Dr. Maisch, Ammerbuch, Germany) was used as an analytical column with “stone-arch” frit . A spray voltage of 2400 V was applied. The injection volume was 5 µL and the flow rate was 500 nL/min. The mobile phases consisted of (A) 0.5% acetic acid and (B) 0.5% acetic acid and 80% acetonitrile. A three-step linear gradient of 5% to 10% B in 5 min, 10% to 40% B in 60 min, 40% to 100% B in 5 min and 100% B for 10 min was employed. The MS scan range was m/z 300–1500. The top ten precursor ions were selected in the MS scan by Orbitrap with R = 60,000, and for subsequent MS/MS scans by ion trap in the automated gain control (AGC) mode where AGC values of 5.00e+05 and 1.00e+04 were set for full MS and MS/MS, respectively. The normalized CID was set to 35.0. A lock mass function was used for the LTQ-Orbitrap XL to obtain constant mass accuracy during gradient analysis .

***Database search and Sequence alignment***

Peptides and proteins were identified by MASCOT v2.2 (Matrix Science, London, U.K.) against the MSU rice protein, cDNA, transcript and genome databases with strict specificity allowing for 1 missed cleavage only. MASCOT identification parameters were Carbamidomethyl (C) as a fixed modification and Acetyl (N-term), Gln->pyro-Glu (N-term Q), Glu->pyro-Glu (N-term E) and Oxidation (M) as partial modifications. The product ion mass tolerance was 0.80 Da, while the precursor ion mass tolerance was 3 ppm. Peptides were rejected if the MASCOT score was below the 99.9% (p value < 0.001) confidence limit based on the “identity” score of each peptide in all database searches. To increase the identification accuracy and peptide specificity, we accepted peptides with at least seven amino acids . Alignment was performed using a local version of NCBI BLAST (blast2seq) Windows version and perl script. We used the default parameters of BLAST. Perl scripts were written for all other data analysis and manipulation steps (see results).

**Supplementary References**

1. Sugiyama N, Nakagami H, Mochida K, Daudi A, Tomita M, Shirasu K, Ishihama Y: **Large-scale phosphorylation mapping reveals the extent of tyrosine phosphorylation in Arabidopsis**. *Molecular systems biology* 2008, **4**:193.

2. Ishihama Y, Rappsilber J, Mann M: **Modular stop and go extraction tips with stacked disks for parallel and multidimensional Peptide fractionation in proteomics**. *Journal of proteome research* 2006, **5**(4):988-994.

3. Rappsilber J, Ishihama Y, Mann M: **Stop and go extraction tips for matrix-assisted laser desorption/ionization, nanoelectrospray, and LC/MS sample pretreatment in proteomics**. *Analytical chemistry* 2003, **75**(3):663-670.

4. Saito H, Oda Y, Sato T, Kuromitsu J, Ishihama Y: **Multiplexed two-dimensional liquid chromatography for MALDI and nanoelectrospray ionization mass spectrometry in proteomics**. *Journal of proteome research* 2006, **5**(7):1803-1807.

5. Wu CC, MacCoss MJ, Howell KE, Yates JR, 3rd: **A method for the comprehensive proteomic analysis of membrane proteins**. *Nature biotechnology* 2003, **21**(5):532-538.

6. Cargile BJ, Bundy JL, Freeman TW, Stephenson JL, Jr.: **Gel based isoelectric focusing of peptides and the utility of isoelectric point in protein identification**. *Journal of proteome research* 2004, **3**(1):112-119.

7. Rappsilber J, Mann M, Ishihama Y: **Protocol for micro-purification, enrichment, pre-fractionation and storage of peptides for proteomics using StageTips**. *Nature Protocols* 2007, **2**(8):1896-1906.

8. Ishihama Y, Rappsilber J, Andersen JS, Mann M: **Microcolumns with self-assembled particle frits for proteomics**. *Journal of chromatography* 2002, **979**(1-2):233-239.

9. Olsen JV, de Godoy LM, Li G, Macek B, Mortensen P, Pesch R, Makarov A, Lange O, Horning S, Mann M: **Parts per million mass accuracy on an Orbitrap mass spectrometer via lock mass injection into a C-trap**. *Molecular and Cellular Proteomics* 2005, **4**(12):2010-2021.

10. Perkins DN, Pappin DJ, Creasy DM, Cottrell JS: **Probability-based protein identification by searching sequence databases using mass spectrometry data**. *Electrophoresis* 1999, **20**(18):3551-3567.

11. Ouyang S, Zhu W, Hamilton J, Lin H, Campbell M, Childs K, Thibaud-Nissen F, Malek RL, Lee Y, Zheng L *et al*: **The TIGR Rice Genome Annotation Resource: improvements and new features**. *Nucleic acids research* 2007, **35**(Database issue):D883-887.

12. Kim W, Silby MW, Purvine SO, Nicoll JS, Hixson KK, Monroe M, Nicora CD, Lipton MS, Levy SB: **Proteomic detection of non-annotated protein-coding genes in Pseudomonas fluorescens Pf0-1**. *PloS one* 2009, **4**(12):e8455.

13. Choudhary JS, Blackstock WP, Creasy DM, Cottrell JS: **Matching peptide mass spectra to EST and genomic DNA databases**. *Trends Biotechnology* 2001, **19**(10 Suppl):S17-22.

14. Altschul SF, Gish W, Miller W, Myers EW, Lipman DJ: **Basic local alignment search tool**. *Journal of molecular biology* 1990, **215**(3):403-410.

15. Tatusova TA, Madden TL: **BLAST 2 Sequences, a new tool for comparing protein and nucleotide sequences**. *FEMS microbiology letters* 1999, **174**(2):247-250.
